# Association of Same-Session Neuroendoscopic Lavage With Ventriculoperitoneal Shunting and Outcomes in Post-hemorrhagic Hydrocephalus: A Retrospective Cohort Study

**DOI:** 10.7759/cureus.99428

**Published:** 2025-12-17

**Authors:** Sait Kayhan, Mehmet C Ezgu, Ecma Yilmaz, Yusuf Izci

**Affiliations:** 1 Department of Neurosurgery, Gülhane Training and Research Hospital, Ankara, TUR; 2 Department of General Practice, Karadeniz Eregli State Hospital, Zonguldak, TUR

**Keywords:** intraventricular hemorrhage, neuroendoscopic lavage, post-hemorrhagic hydrocephalus, prematurity, ventriculoperitoneal shunt

## Abstract

Background

We aimed to assess whether adding neuroendoscopic lavage (NEL) at the time of ventriculoperitoneal shunting (VPS) provides postoperative advantages in infants with post-hemorrhagic hydrocephalus (PHH).

Materials and methods

We retrospectively reviewed 73 infants with PHH requiring permanent cerebrospinal fluid (CSF) diversion. Forty-two underwent combined NEL + VPS, while thirty-one underwent VPS alone. Demographic data, intraventricular hemorrhage (IVH) grade, operative characteristics, postoperative complications, reoperation rate, time to revision, and mortality were compared between groups. The primary outcome was reoperation rates. Secondary outcomes included complications and mortality.

Results

Baseline demographic and radiographic parameters were similar between groups. Mean operative duration was longer in the NEL + VPS group (78.4 ± 13.2 min vs. 52.6 ± 5.2 min), with comparable intraoperative blood loss (8.21 vs. 8.10 mL). Reoperation was required in nine (21.4%) patients in the NEL + VPS group compared with 17 (54.8%) in the VPS-only group. Overall complication rates and mortality were numerically lower in the NEL + VPS cohort (complications: 40.5% vs. 61.3%; mortality: 14.3% vs. 32.3%), although these differences did not reach statistical significance. Time to reoperation was shorter in the NEL + VPS group (25.1 vs. 53.5 days), and follow-up duration was comparable between cohorts. The primary outcome was reoperation rates. Secondary outcomes included complications and mortality.

Conclusions

This study presents a complementary perspective on the role of NEL by evaluating its use as a same-session adjunct to definitive VPS, rather than as a staged or shunt-sparing intervention, thereby reflecting a pragmatic surgical strategy in shunt-eligible infants. Our findings suggest that same-session NEL may enhance shunt performance and reduce revision requirements in infants who already require permanent CSF diversion. Further prospective multicenter studies are needed to better define optimal patient selection and long-term outcomes.

## Introduction

Post-hemorrhagic hydrocephalus (PHH) is a downstream consequence of intraventricular hemorrhage (IVH), seen in both preterm and term infants, particularly those with extremely low birth weight. The primary effect of hemorrhage on intracranial pressure (ICP) and the secondary effects of neurotoxic byproducts from blood degradation cause deterioration in neurological and functional development, especially when combined with the immaturity of the preterm immune system [1].

Across neurosurgical studies and meta-analyses, the optimal treatment algorithm for PHH associated with IVH has been discussed; however, no consensus has been reached. Accordingly, both traditional surgical interventions - such as temporizing cerebrospinal fluid (CSF) diversion and permanent shunting - and neuroendoscopic techniques have been applied in the management of PHH [2,3]. Although permanent shunting is generally considered unfavorable in premature infants, particularly due to extremely low birth weight and immaturity, it becomes necessary when temporary CSF management strategies fail to adequately control ventricular dilatation and ICP [4,5].

Intraventricular lavage using an endoscope, also known as neuroendoscopic lavage (NEL), has gained increasing interest in recent years [6-8]. This approach allows intraoperative clearance of hemorrhagic CSF, which may lead to restored CSF circulation and a reduced burden of blood degradation products and protein load [9,10]. Nevertheless, repeated intraventricular lavage may be required, particularly in severe IVH, such as Grade III or IV IVH according to the Papile classification system [11-13]. This requirement is undesirable because of prolonged operative time and increased anesthetic exposure. While intraventricular lavage has gained increasing attention and has been reported to be safe in several series, management strategies preceding permanent shunting - such as external ventricular drainage (EVD) or serial lumbar punctures - vary considerably across institutions. Considering that NEL appears to decrease shunt dependency and shunt-related complications in selected cases [2,10,14], its therapeutic value when integrated into a permanent shunting strategy remains insufficiently defined.

This study aims to determine whether the addition of NEL to definitive ventriculoperitoneal shunting (VPS) improves postoperative outcomes compared with shunting alone in infants with PHH. The primary outcome of this study was to evaluate whether the addition of NEL to VPS reduces the need for shunt reoperation in infants with PHH. Secondary outcomes included comparisons of overall complication rates, mortality, operative parameters, and time to reoperation between NEL + VPS and VPS-only cohorts.

## Materials and methods

Patient selection and study design

The present study was conducted through a retrospective evaluation of 73 infants who developed persistent PHH necessitating permanent CSF diversion following IVH between 2010 and 2020 at our institution. These 73 infants represent the subset of IVH patients who reached the clinical and radiological threshold for definitive shunting, rather than all PHH with IVH cases. Of these, 42 underwent combined NEL and VPS during the same session, while 31 underwent VPS alone. Clinical files were reviewed for demographic characteristics, radiographic measurements, and detailed operative and clinical documentation.

PHH was defined as progressive ventricular dilatation after IVH, documented on serial cranial ultrasonography (cUS). IVH severity was graded according to the Papile classification system (Grades I-IV) [13]. Ventricular size was assessed using the ventricular index (VI), anterior horn width (AHW), and thalamo-occipital distance (TOD), adjusted for gestational age-specific reference values. In line with recent consensus recommendations [15], no single absolute threshold was used. Instead, the decision to proceed with permanent CSF diversion was based on a combination of (i) progressively increasing ventricular dimensions on serial cUS, (ii) increased head circumference growth (>1.25 cm/week), and/or (iii) clinical signs of elevated ICP, including bulging fontanelle, apneic episodes, irritability, feeding impairment, or bradycardia.

The choice of surgical strategy reflected institutional practice and multidisciplinary clinical judgement. Within this clinically homogeneous cohort of shunt-eligible PHH infants, NEL + VPS was preferentially chosen for patients with denser intraventricular clot burden or more rapid ventricular enlargement, rather than for anatomical complexity, while others underwent primary VPS alone. The decision-making process incorporated dynamic clinical factors not fully captured by static baseline measurements. This approach allowed clinical equipoise to be maintained while tailoring surgical strategy to individual patient evolution. No endoscopic septostomy or additional intraventricular compartment fenestration was performed in this cohort. Endoscopic third ventriculostomy (ETV) was not performed, as PHH in premature infants is predominantly related to impaired CSF absorption rather than obstructive pathology. Moreover, intraventricular blood products and inflammatory debris have been shown to negatively affect ETV success rates in this population [12]. Therefore, VPS was preferred as the definitive treatment strategy in this cohort.

Clinical outcomes were predefined prior to analysis. Shunt reoperation was defined as any unplanned surgical intervention requiring revision, replacement, or repositioning of the VPS system for mechanical dysfunction, obstruction, or infection. Complications were recorded as postoperative events, including shunt infection, seizure, or secondary intracranial hemorrhage, based on clinical assessment and radiological confirmation when applicable. Mortality was defined as surgery-related death during the follow-up period. Follow-up consisted of routine clinical examinations and cUS during the early postoperative period, with additional imaging and interventions performed as clinically indicated. Across all 73 cases, complete clinical and radiological datasets were available, with no missing variables for analysis.

Surgical technique

All surgical procedures were performed under general anesthesia. Patients were placed in the supine position, with the head slightly elevated and gently rotated to facilitate access to the planned entry site. Particular attention was given to maintaining normothermia throughout the operation, especially in premature and low-birth-weight infants. A meticulous microsurgical technique was adopted to protect the soft tissues and fragile skin of these vulnerable patients.

VPS-Only

In both groups, ventricular access was obtained through the most enlarged lateral ventricle, as determined by preoperative neuroimaging, using standard frontal entry points such as Kocher’s or Dandy’s point, depending on individual ventricular anatomy. A standard curvilinear frontal skin incision was made, followed by burr-hole creation. After dural opening, the ventricle was cannulated using anatomical landmarks.

Right-angled or straight CSF reservoir systems (Medtronic Inc., Goleta, CA, USA) were used for ventricular access. Reservoir patency was routinely assessed intraoperatively by gentle tapping using a small-gauge needle, when clinically indicated. For shunt implantation, position-sensitive Christoph Miethke® paediGAV valves (Aesculap, Tuttlingen, Germany) were utilized. The opening pressures of these valves were 4 or 9 cm H₂O in the horizontal position and 24 cm H₂O in the vertical position. The distal catheter was subcutaneously tunneled and introduced into the peritoneal cavity through a small abdominal incision.

NEL + VPS

In patients undergoing adjunct endoscopic lavage, ventricular access was obtained through a frontal burr hole, with the approach tailored to ventricular anatomy; in selected cases, an occipital trajectory may also be considered. Following dural opening, a small corticotomy was performed using bipolar coagulation. Intraventricular hematoma remnants and debris were visualized using a Karl Storz 0° or 30° rigid endoscope (Karl Storz GmbH & Co. KG, Tuttlingen, Germany).

**Figure 1:**
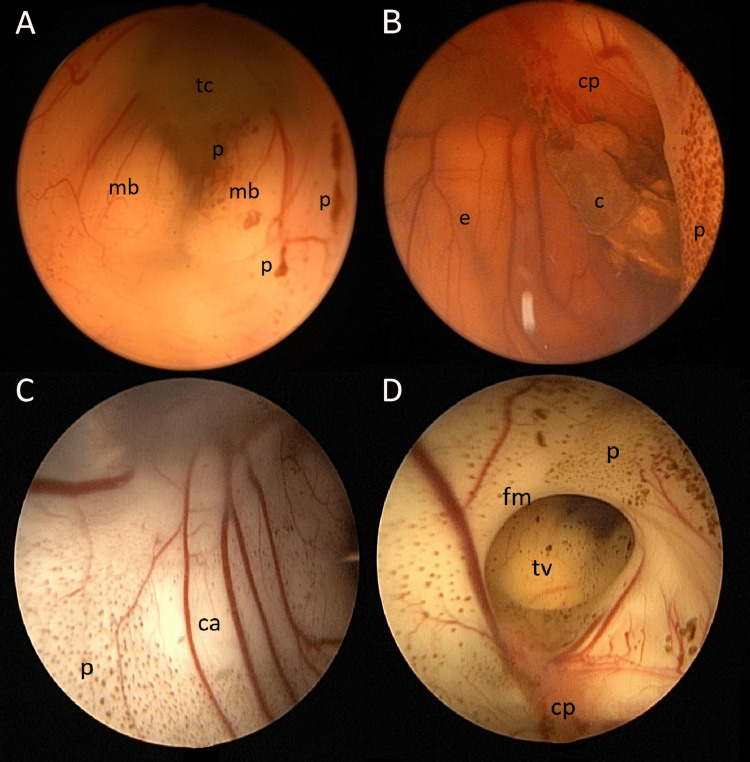
Endoscopic intraoperative views in infants with PHH (A) Third ventricle demonstrating mamillary bodies (mb), tuber cinereum (tc), and posthemorrhagic particles (p). (B) Right lateral ventricle showing ependyma (e), choroid plexus (cp), an adherent coagulum (c), and widespread particulate debris (p). (C) Interventricular septum covered with posthemorrhagic particles (p), with areas cleared by irrigation (ca). (D) Right lateral ventricle with visible foramen of Monro (fm), choroid plexus (cp), posthemorrhagic particles (p), and floor of the third ventricle (tv). PHH: Post-hemorrhagic hydrocephalus

Continuous irrigation was performed using warmed Ringer’s lactate solution (35-37°C), with an approximate total volume of 2-2.5 L per procedure, adjusted according to intraventricular clarity and hemodynamic tolerance. Under continuous irrigation, hematoma residues, clots, and hemosiderin-laden debris were gently mobilized and aspirated. The ventricular cavities were systematically inspected to optimize clearance and ensure restoration of CSF pathways (Figure 1). After completion of lavage, the endoscope was removed, and VPS implantation was performed during the same session, using the technique described above, including ventricular access systems and CSF reservoirs. CSF samples were obtained from all patients as a routine part of the intraoperative procedure in both groups.

Postoperative management

Postoperatively, all neonates were monitored in the neonatal intensive care unit during the immediate recovery period, including neurological surveillance, ICP-related clinical monitoring, and routine laboratory tracking. Routine follow-up included serial white blood cell (WBC) and C-reactive protein (CRP) measurements, CSF analysis and culture when indicated, and inspection of the shunt tract and scalp integrity.

Postoperatively, patients were closely monitored for potential complications, including infection, seizure activity, and secondary hemorrhage. In cases where shunt infection was suspected, management proceeded according to standard neurosurgical infection-control principles, including prompt shunt removal, external CSF diversion via EVD, and targeted intravenous antimicrobial therapy in accordance with CSF culture results, followed by delayed re-shunting once microbiological clearance was confirmed. Anticonvulsant medication was not administered prophylactically, but was initiated in response to any clinical seizure episode or suspicious paroxysmal manifestations. Given the often subtle nature of neonatal seizures, monitoring specifically included vigilance for apnea, lip smacking, ocular deviation, autonomic fluctuations, and other non-convulsive presentations. Similarly, vigilance for secondary hemorrhage was maintained through early postoperative neuroimaging (cUS or CT) to verify catheter placement and exclude interval bleeding. Any concerning neurological change, such as new-onset irritability, altered consciousness, apneic spells, or sudden ventricular expansion on cUS, prompted expedited reassessment for potential rebleeding.

Ethical approval and statements

The Local Institutional Review Board approved the study (date: December 17, 2020; No: 2020-419). This study was conducted in accordance with Strengthening the Reporting of Observational Studies in Epidemiology (STROBE) guidelines and the Helsinki Declaration [16]. We would also like to declare that this study is derived from the corresponding author’s master’s thesis titled “Evaluation of Surgical Techniques and Outcomes in Pediatric Patients with Hydrocephalus,” completed in 2021 at the Gülhane Training and Research Hospital, Department of Neurosurgery.

The present manuscript represents an expanded and reanalyzed version of a specific subgroup originally included in that thesis. For the current study, the dataset was broadened to include additional eligible patients operated on by other attending neurosurgeons in the same clinic, thereby increasing the sample size and strengthening the statistical validity of subgroup comparisons. All contributing surgeons granted permission for the use of their cases, and the study was conducted in accordance with institutional ethical regulations.

Statistical analysis

Continuous variables were assessed for normality using the Shapiro-Wilk test. Normally distributed variables were expressed as mean ± standard deviation (SD) and compared using the independent samples t-test. Non-normally distributed variables were summarized as median (minimum-maximum) and compared using the Mann-Whitney U test.

Categorical variables were presented as frequencies and percentages, and differences between groups were compared using the Chi-square test or Fisher’s exact test, where appropriate. For variables with two-level binomial outcomes, the association between surgical modality and outcome was evaluated using contingency analysis.

A significance level of p < 0.05 was accepted as statistically significant in all analyses. IBM SPSS Statistics for Windows, Version 25 (Released 2017; IBM Corp., Armonk, NY, USA), a statistical package program was used for data analysis.

## Results

A total of 73 infants with PHH underwent definitive surgical treatment, with 42 receiving combined NEL + VPS (Group A) and 31 receiving primary VPS-only (Group B). Baseline demographic parameters, including sex distribution (female: 23/42 (54.8%) vs. 16/31 (51.6%); male: 19/42 (45.2%) vs. 15/31 (48.4%)), gestational age at birth (29 + 5 vs. 29 + 6), age at operation (31 + 2 vs. 32 + 0), weight at birth (1411 g vs. 1579 g), and weight at operation (1680 g vs. 1876 g), showed no statistically significant differences between groups, indicating good baseline comparability. The distribution of IVH severity (Grade II-IV) was also similar between the two cohorts (Grade II: 13 (31.0%) vs. 9 (29.0%); Grade III: 15 (35.7%) vs. 11 (35.5%); Grade IV: 14 (33.3%) vs. 11 (35.5%)). No patients in either group had Grade I IVH (0%) (Table 1).

**Table 1:** Baseline characteristics of the patients *These parameters are reported as median values. NEL: Neuroendoscopic lavage; VPS: Ventriculoperitoneal shunt; IVH: Intraventricular hemorrhage

	NEL + VPS (Group A) (n = 42)	VPS-only (Group B) (n = 31)
Sex (n, %)		
Female	23 (54.8%)	16 (51.6%)
Male	19 (45.2%)	15 (48.4%)
Age at birth, weeks and days*	29+5 (24+5 - 35+1)	29+6 (24+6 - 34+3)
Weight at birth, g	1411 ± 387 (750-2350)	1579 ± 389 (900-2100)
Age at operation, weeks and days*	31+2 (25+5 - 40+5)	32+0 (28+2 - 37+3)
Weight at operation, g	1680 ± 425 (820-3300)	1876 ± 339 (1120-2800)
IVH Grades (n,%)		
Grade I	0 (0%)	0 (0%)
Grade II	13 (31.0%)	9 (29.0%)
Grade III	15 (35.7%)	11 (35.5%)
Grade IV	14 (33.3%)	11 (35.5%)
Time for operation, days	15.2 ± 9.7	14.8 ± 8.4
Comorbid diseases, no, (n, %)	2 (4.8%)	3 (9.6%)
Ventricular index, mm	18.4 ± 3.5 (12-27)	19.8 ± 4.6 (12-30)
Anterior-horn width, mm	5.9 ± 2.0 (3-11)	6.5 ± 2.5 (3-13)
Thalamo-occipital distance, mm	26.6 ± 4.7 (18-36)	28.3 ± 6.1 (18-40)

Preoperative ventricular measurements were comparable between groups, although Group B showed slightly higher mean AHW (6.5 vs. 5.9 mm) and TOD (28.3 vs. 26.6 mm); these differences did not reach statistical significance. Time from birth to surgery was similar between groups (15.2 vs. 14.8 days) (Table 1).

Overall, the NEL + VPS and VPS-only cohorts were statistically comparable at baseline across all demographic, clinical, and radiological parameters (all p > 0.05).

Intraoperatively, the mean operation time was significantly longer in Group A (78.4 ± 13.2 min, p < 0.001) compared with Group B (52.6 ± 5.2 min). Blood loss was similar between groups (8.21 mL vs. 8.10 mL). Postoperatively, reoperation was required in nine of 42 patients (21.4%) in Group A and 17 of 31 patients (54.8%) in Group B (χ² = 7.29, df = 1, p = 0.0019, Cramer’s V = 0.32). Notably, the time to reoperation was shorter in Group A (25.1 days) compared with Group B (53.5 days) (Table 2).

**Table 2:** Operative and clinical parameters of the patients NEL: Neuroendoscopic lavage; VPS: Ventriculoperitoneal shunt; NICU: Neonatal intensive care unit

	NEL + VPS (Group A) (n = 42)	VPS-only (Group B) (n = 31)	p-value
Operation time, min	78.4 ± 13.2 (47-110)	52.6 ± 5.2 (43-63)	<0.001
Blood loss, mL	8.21 ± 3.37 (3-14)	8.10 ± 3.17 (3-13)	0.26
Reoperation (n, %)	9 (21.4%)	17 (54.8%)	0.02
Reoperation time, days after initial surgery	25.1 ± 11.7 (9-45)	53.5 ± 44.0 (10-180)	0.25
Complication (n, %)	17 (40.5%)	19 (61.3%)	0.13
Length of stay in NICU, days	82.0 ± 29.1 (34-135)	82.9 ± 29.5 (39-134)	0.54
Mortality (n, %)	6 (14.3%)	10 (32.3%)	0.12
Follow-up time, months	35.7 ± 15.7 (3-60)	31.4 ± 19.4 (3-59)	0.42

Overall postoperative complication rates were 40.5% (17 of 42 patients) in Group A and 61.3% (19 of 31 patients) in Group B (χ² = 2.31, df = 1, p = 0.128, Cramer’s V = 0.18). Mortality was numerically lower in Group A compared with Group B (6/42 (14.3%) vs. 10/31 (32.3%), χ² = 2.40, df = 1, p = 0.121, Cramer’s V = 0.18); however, neither the difference in complication rates nor mortality reached statistical significance. Accordingly, these findings should be interpreted as numerical trends rather than statistically significant differences. Mean follow-up duration was comparable (35.7 vs. 31.4 months) (Table 2).

## Discussion

High-grade IVH frequently progresses into PHH, significantly affecting neurological development and long-term functionality, with reported outcomes ranging from normal neurodevelopment to motor impairments, mixed-type cerebral palsy, cognitive deficits, and broader neurocognitive disability [1,17]. While a consensus regarding optimal PHH treatment is lacking, the most common strategy relies on staged CSF management, beginning with temporary procedures such as tapping, EVD, or ventriculosubgaleal shunts [1-3]. These temporizing approaches are often chosen due to the physiological immaturity of premature infants, particularly regarding immune function and peritoneal absorption capacity [17]. Although even in such cases, definitive shunting eventually becomes necessary in the majority of patients, as temporary methods only delay, but do not eliminate, the need for permanent CSF diversion [4,5,18].

Several studies have shown that early ventricular washing reduces shunt dependency when used in earlier disease stages [2,6,8-10]. NEL represents a minimally invasive evolution of earlier intraventricular blood clearance strategies, such as drainage, irrigation, and fibrinolytic therapy (DRIFT), by directly targeting IVH debris in a more focused and controlled manner [18]. A more recent meta-analysis concluded that ventricular lavage significantly decreases shunt dependency compared to standard management [2]. Beyond single-center experiences, contemporary PHH management is now increasingly informed by large-scale prospective data initiatives. The TROPHY registry represents an ongoing international multicenter effort that systematically captures real-world surgical management patterns and outcomes in neonates with PHH, although comprehensive pooled results have not yet been reported [19]. Similarly, the ENLIVEN-UK randomized controlled trial is actively evaluating endoscopic lavage in a standardized, protocol-driven manner across multiple centers, with final outcome data still pending [20]. However, these findings primarily apply to populations in which permanent shunting was not yet inevitable. In this context, we specifically evaluated infants who were eligible for permanent shunt dependence, enabling a focused assessment of NEL as an intraoperative adjunct.

A key finding in our cohort is the significantly lower reoperation rate observed in the NEL + VPS group compared with the VPS-only group (21.4% vs. 54.8%), suggesting that intraventricular blood clearance may improve long-term shunt functionality. This observation aligns with prior reports demonstrating a reduced shunt revision burden following NEL [6-8]. From a functional point of view, NEL facilitates early removal of intraventricular debris, hemoglobin degradation products, and inflammatory mediators, thereby improving catheter patency and stabilizing CSF dynamics [6-8,10,21,22]. While some studies have additionally suggested potential benefits, such as reduced multiloculated hydrocephalus formation or delayed shunt requirement [1,3,5], these effects were not the primary focus of the present cohort. Importantly, although many previous investigations emphasized shunt avoidance or delay, our findings demonstrate that adjunct NEL may remain beneficial even in patients for whom permanent shunting is inevitable.

Within this context, the shorter interval to reoperation observed in the NEL + VPS group (25.1 vs. 53.5 days) should be interpreted not as a marker of inferior shunt performance but as a complementary manifestation of the same underlying mechanism. By restoring intraventricular conditions and CSF circulation earlier, NEL may unmask shunt malfunction sooner once compensatory pathways recover, allowing obstruction to become clinically apparent and addressed in a timely manner. In contrast, persistent intraventricular debris in VPS-only patients may contribute to a more gradual and insidious decline in shunt performance, resulting in delayed detection of failure. Thus, the combination of a lower overall revision burden, together with a shorter time to reoperation in the NEL + VPS group, may reflect earlier physiological stabilization and prompt recognition of malfunction rather than diminished shunt durability. To our knowledge, this temporal pattern has not been previously described and represents a novel observation of the present study. However, alternative explanations should also be considered, including procedural bias and surveillance bias, although both groups were managed within the same department under comparable perioperative and follow-up protocols to minimize bias.

We observed numerically lower overall complication and mortality rates in the NEL cohort compared with the VPS-only group (complications: 40.5% vs. 61.3%; mortality: 14.3% vs. 32.3%); however, neither difference reached statistical significance. Accordingly, no causal inference can be drawn, and these findings should be interpreted with caution and regarded as exploratory. The lower complication profile observed may be related to improved intraventricular conditions, such as reduced catheter obstruction and a lower tendency toward subsequent shunt revisions, although baseline neonatal vulnerability likely plays a substantial role. Previous studies have demonstrated that infection, rebleeding, seizure incidence, and shunt-related outcomes are strongly influenced by prematurity-associated systemic comorbidities rather than surgical technique alone [1,7,12,17]. In this context, Abdelmageed et al. reported that conditions such as necrotizing enterocolitis and hyaline membrane disease were more strongly associated with shunt-related outcomes than the chosen surgical pathway [7]. Taken together, these findings suggest that, while NEL may improve intraventricular clearance and local CSF dynamics, it cannot fully offset the risks driven by systemic immaturity [23]. The numerically lower mortality observed in the NEL + VPS group may reflect a potential stabilizing effect on intracranial homeostasis and systemic stress [14], but this hypothesis requires confirmation in larger, adequately powered prospective studies. It should also be acknowledged that NEL involves intraventricular manipulation and therefore carries a theoretical risk of procedure-related intraventricular bleeding, particularly in premature infants with fragile ventricular walls. In our cohort, no clinically significant procedure-related hemorrhage was observed. Nevertheless, this potential risk should be considered when interpreting the safety profile of the technique.

Blood loss was nearly identical between groups (8.21 vs. 8.10 mL), indicating that lavage does not elevate intraoperative risk, despite extended procedural duration [9,21]. Operation time was significantly longer in the NEL + VPS group (78.4 vs. 52.6 minutes); however, this prolonged exposure did not correspond to worse immediate postoperative outcomes, suggesting acceptable procedural tolerability.

The findings of this study may also have implications for centers without neuroendoscopic capability. While NEL requires specific equipment and expertise, the observed benefits in our cohort were primarily related to reduced reoperation rates rather than secondary outcomes, such as overall complication or mortality rates, which did not differ significantly between groups. In centers where endoscopic lavage is not available, meticulous surgical technique, careful shunt placement, and close postoperative surveillance remain essential, and treatment strategies should be adapted to institutional resources and expertise.

Limitations of the study

This study has several limitations. First, its retrospective, single-center design introduces inherent risks of selection bias and limits the generalizability of the findings. Although the sample size was expanded by incorporating cases operated by multiple surgeons, the overall cohort remains relatively small, limiting statistical power, particularly for subgroup comparisons. Consequently, the limited number of outcome events precluded reliable multivariable analyses, as such modeling would carry a high risk of overfitting and unstable estimates. For this reason, the analyses were restricted to univariable comparisons, and all findings should be interpreted as exploratory. Second, the timing of permanent CSF diversion was based primarily on clinical progression and serial ultrasonographic findings. Objective thresholds proposed in the literature, such as ventricular ratio cutoffs, persistent reservoir tapping, or predefined weight criteria, were not formally applied, which may limit the generalizability of our results. Third, surgical decision-making - specifically the choice between NEL + VPS versus VPS-only - was not randomized but guided by clinical judgment, which may have introduced treatment-selection bias related to illness severity or clot burden. Fourth, long-term neurodevelopmental outcomes were not evaluated, limiting the ability to determine whether the observed short- and mid-term benefits of NEL translate into improved functional or cognitive trajectories.

## Conclusions

The present study focuses on the effect of NEL in infants already requiring permanent shunting. Our findings suggest that, in this context, the primary benefit of adjunct NEL may lie in reducing reoperation rates and optimizing shunt performance. Although overall complication and mortality rates were numerically lower in the NEL cohort, these differences did not reach statistical significance and should be interpreted with appropriate statistical caution. Accordingly, NEL may serve as a valuable intraoperative adjunct in permanently shunted PHH patients, without introducing additional surgical risk. Prospective, multicenter studies are warranted to further define its long-term role and optimal clinical indications.
